# Mapping the research trends and hotspots of exercise and nutrition in diabetes: a bibliometric and visual analysis (2005–2025)

**DOI:** 10.3389/fnut.2025.1680190

**Published:** 2025-10-09

**Authors:** Zhouluo Wang, Yuxuan He, Jingyu Wang, Yi Sun

**Affiliations:** ^1^School of Philosophy and Sociology, Jilin University, Changchun, China; ^2^School of Education and Arts, Jiujiang Polytechnic University of Science and Technology, Jiujiang, China; ^3^Department of Sport Leisure, Sungshin Women's University, Seoul, Republic of Korea; ^4^Physical Education College, Jilin University, Changchun, China

**Keywords:** diabetes, exercise intervention, nutrition therapy, bibliometrics, visualization

## Abstract

**Objectives:**

Despite the growing interest in exercise and nutrition as key strategies for diabetes prevention and management, a comprehensive bibliometric assessment of this field remains lacking. This study aims to map the research landscape, identify research trends and hotspots to inform future academic inquiry and clinical practice.

**Methods:**

As of July 3, 2025, publications on exercise and nutrition in diabetes from 2005 to 2025 were retrieved from the Web of Science Core Collection and Scopus databases. The bibliometric and visual analysis was conducted using R software, VOSviewer, and CiteSpace.

**Results:**

Trends in annual publication outputs have shown a consistent upward trajectory from 2005 to 2025. The United States led in both research output and institutional prominence. China, South Korea, Australia, and Canada also emerged as key contributors, and European countries functioned as major collaborative centers. *Nutrients* and *the American Journal of Clinical Nutrition* ranked among the most prolific and frequently cited sources in the field. Co-citation, burst detection, keyword frequency, clustering, and thematic evolution collectively revealed three major thematic domains: (1) lifestyle interventions in diabetes focusing on different exercise types, nutritional approaches, and their combinations; (2) management of long-term diabetic complications through physical activity and dietary approaches; and (3) population-specific strategies for older adults, children, and women with and at risk of diabetes. Across these themes, studies have prominently highlighted mechanistic insights, therapeutic efficacy, evidence-based guidelines, risk management, and adherence.

**Conclusion:**

Over the past two decades, attention to this field has steadily increased, with strong collaboration established among countries, institutions, and journals. Emerging research trends in exercise and nutrition in diabetes are shifting toward a life course–oriented paradigm, personalized self-management support, and more innovative, adaptable intervention formats tailored to accommodate modern lifestyles.

## 1 Introduction

Diabetes is a group of metabolic disorders characterized by hyperglycemia resulting from impairment of insulin secretion, defects in insulin action, or a combination of both (1). The vast majority of diabetes cases are classified into two main etiopathogenetic types: type 1 diabetes (T1D), caused by a deficiency in β-cell function, and type 2 diabetes (T2D), resulting from insulin resistance and inadequate insulin secretion (2). Uncontrolled diabetes can lead to acute, life-threatening conditions such as diabetic ketoacidosis and hyperglycemic hyperosmolar nonketotic syndrome (3, 4). Moreover, chronic microvascular and macrovascular complications include retinopathy, nephropathy, neuropathy, as well as cardiovascular and cerebrovascular diseases (5). These pathologies contribute to the dysfunction of cells, tissues, and organ systems, particularly affecting the eyes, kidneys, nervous, heart, and blood vessels (1, 6, 7). According to the International Diabetes Federation (IDF) 2025 report, 589 million adults aged 20–79 were living with diabetes, accounting for roughly one in nine people globally, and this figure is projected to climb to 853 million by 2050. In 2024, diabetes-related causes were responsible for 3.4 million deaths. The economic burden is substantial as well, with direct health expenditures related to diabetes exceeding USD 1 trillion for the first time, representing a 338% increase over the past 17 years (8). Thus, diabetes constitutes a major global health challenge with high morbidity, mortality, and financial demands, necessitating early preventive strategies and effective long-term management (9).

Current diabetes treatments include pharmacological, surgical, and lifestyle interventions (10, 11). With a growing number of pharmacological agents available, concerns over potential side effects and healthcare costs have been increasing (12). Alternatively, appropriate exercise and nutritional modifications can improve insulin sensitivity and glycemic control, reduce medication reliance, and offer effective, low-cost strategies to prevent complications (13–15). As demonstrated, physical activity is essential for glycemic management and overall health in individuals with diabetes and prediabetes (16). In T1D, combined aerobic and acute resistance exercise has been shown to enhance glycemic control and support muscle health through activation of muscle signaling pathways involved in substrate metabolism and anabolic adaptations (17). In T2D, long-term exercise has been reported to improve common metabolic abnormalities and their complications, with specific benefits for glycemic control, cardiovascular risk, lipid profile, blood pressure, and potentially improved fibrinolytic function (18). Meanwhile, nutrition therapy remains a fundamental pillar of diabetes prevention and management (19). Several studies have demonstrated that increasing dietary fiber intake and adjusting macronutrient composition can improve glycemic control and reduce arterial stiffness (20), while adopting low glycemic index (GI) diets can facilitate weight loss and enhance insulin action and glucose tolerance (15). Moreover, nutritional supplementation, such as zinc, chromium, magnesium, selenium, vitamins, arginine, and glutamine, may further support diabetes management (21, 22).

Notably, the interaction between exercise and nutrition in diabetes has received considerable attention, as combined interventions have shown promising metabolic benefits. In a randomized controlled trial, significant improvements in fasting glucose were observed exclusively in the exercise and diet group, while the exercise-only group showed no change. Moreover, the combined interventions led to a 188% to 269% greater improvement in insulin sensitivity, as assessed by the Matsuda index, compared to exercise alone (23). Exercise training along with nutrition intake has been shown to synergistically promote skeletal muscle mass by reducing muscle protein breakdown, increasing protein synthesis, and improving amino acid availability, thereby lowering the risk of diabetes (24). Likewise, carbohydrate restriction may support sustained weight loss and improve metabolic outcomes, and when integrated with increased physical activity, it may serve as an important contributor to diabetes remission (25). Furthermore, improvements in brain insulin sensitivity, which influence appetite regulation and energy fluxes, have been observed when physical activity is paired with a low-fat diet, potentially benefiting mental and cognitive function in individuals with diabetes (26). Although several experimental studies, systematic reviews and meta-analyses have investigated the role of exercise and nutrition in diabetes, there remains a lack of comprehensive analyses focusing on research hotspots and frontiers, which hinders a deeper understanding of the field's current trajectory and future directions.

Bibliometrics, as a quantitative and science-mapping approach, enables the systematic analysis of publications, authors, institutions, countries, sources, keywords, and citations within specific research domains (27). A well-conducted bibliometric study can help researchers gain a one-stop overview of the current research landscape, identify knowledge gaps, generate novel investigation ideas, and position future contributions for both basic research and clinical applications (28). Thus, employing VOSviewer, R software, and CiteSpace to visually map the knowledge evolution, intellectual structure, research hotspots, and emerging trends in the field of exercise and nutrition in diabetes, this study aims to equip researchers and clinicians with valuable insights and establish a robust basis for further theoretical and practical advances.

## 2 Materials and methods

### 2.1 Data collection

The data were retrieved from the Web of Science Core Collection (WoSCC) and Scopus databases, and the period was set from January 1, 2005, to July 3, 2025. WoSCC is widely regarded as the world's most trusted citation database, providing consistent, accurate, and comprehensive indexing that facilitates robust research discovery (29). Scopus, a leading abstract and citation database offering curated research and enriched scholarly literature, was also consulted. Together, these two world-class databases, both adhering to rigorous standards of content selection and maintenance, ensured the rigor and inclusiveness of the study dataset (30). Search strategies included the following terms: [TS = (diabetes AND (exercise OR “physical activity”) AND nutrition*)]. The publication types were limited to “Article” and “Review,” and the language was restricted to “English.” The period was from 2005 to 2025. After excluding irrelevant and duplicate documents, a total of 4,793 papers were obtained from WoSCC and 9,187 publications from Scopus. To maximize data completeness, data from WoSCC and Scopus were exported in plain text and csv formats, respectively, with full records and cited references included. Detailed information on the search strategy and inclusion criteria is available in Supplementary material 1.

### 2.2 Data analysis

This study employed three key software tools: R software (Version 4.2.2), VOSviewer (Version 1.6.20), and CiteSpace (Version 6.4.R1), each serving diverse and comprehensive analytical purposes. Bibliometrix, an R-based tool capable of rapidly processing data and performing science mapping (31), was used in this study to calculate fundamental bibliometric metrics and construct a thematic evolution chart. Additionally, graphical visualizations, such as the annual publication trends and leading sources by publication and citation counts, were generated using the ggplot2 package within RStudio. VOSviewer, a Java-based software specialized in constructing and visualizing bibliometric networks (32), was applied to visualize country and institutional co-authorship networks, source co-citation analysis, and keyword co-occurrence. CiteSpace, which incorporates mathematical modeling and statistical algorithms (33), was employed to detect research hotspots and emerging frontiers through its burst detection function. The integration of these three tools allowed us to leverage their complementary strengths, combining statistical rigor, intuitive visualization, and frontier detection into a multidimensional framework that enhanced methodological robustness and enabled a broader analytical perspective for bibliometric analysis (34, 35).

## 3 Results

### 3.1 Overview of selected studies on exercise and nutrition in diabetes

**Figure 1:**
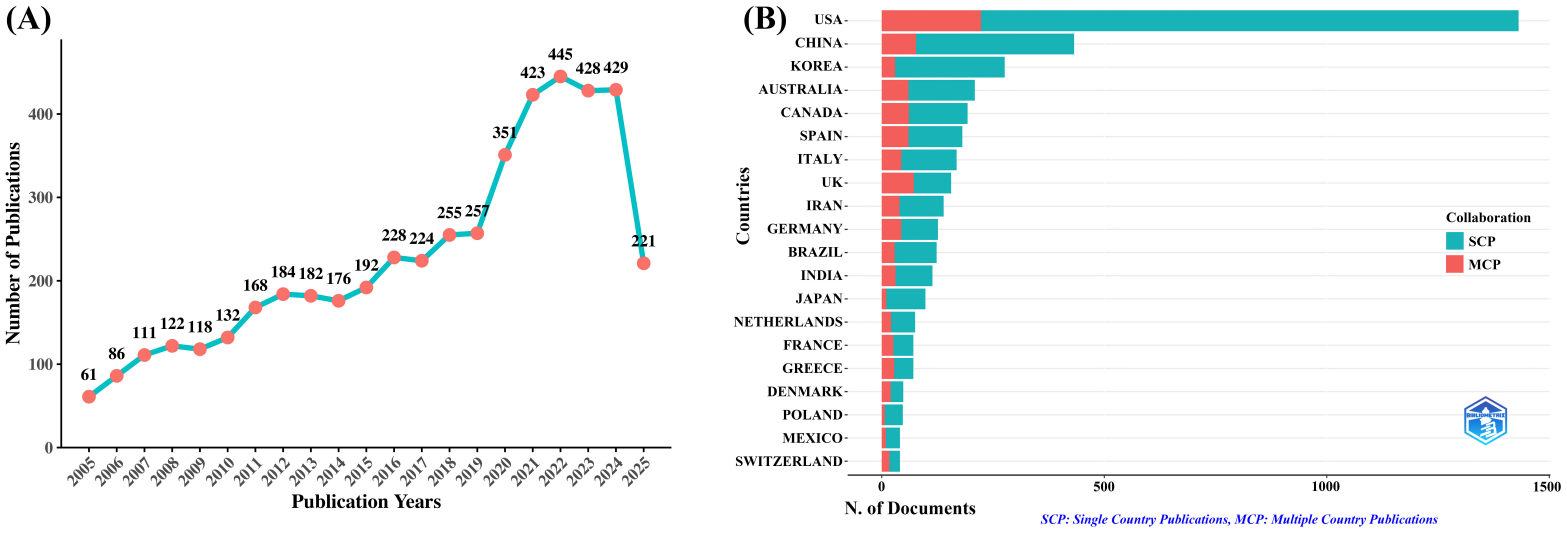
Trends in annual publication outputs on exercise and nutrition in diabetes from 2005 to 2025. **(A)** Trends of annual publication outputs. **(B)** Distribution of corresponding authors countries and cooperation.

A total of 4,793 unique publications were retrieved from the WoSCC database after deduplication. As shown in Figure 1A, the number of publications concerning exercise and nutrition in diabetes has exhibited a consistent upward trajectory from 2005 to 2024. A significant surge in publications occurred between 2019 and 2021, followed by a sustained high output, with each year since 2020 surpassing 400 articles. Similarly, the Scopus database yielded 9,187 unique records after deduplication, with a consistent growth trend observed in Supplementary Figure 1, confirming the increasing scholarly interest in the role of exercise and nutrition in diabetes. Notably, 2024 saw the highest number of annual publications, with 1,049 articles, indicating a peak in research activity. Additionally, as of July 3, 2025, there has been continued academic focus on this critical area, with the WoSCC database recording 221 new publications and Scopus including 668 new articles.

**Figure 2:**
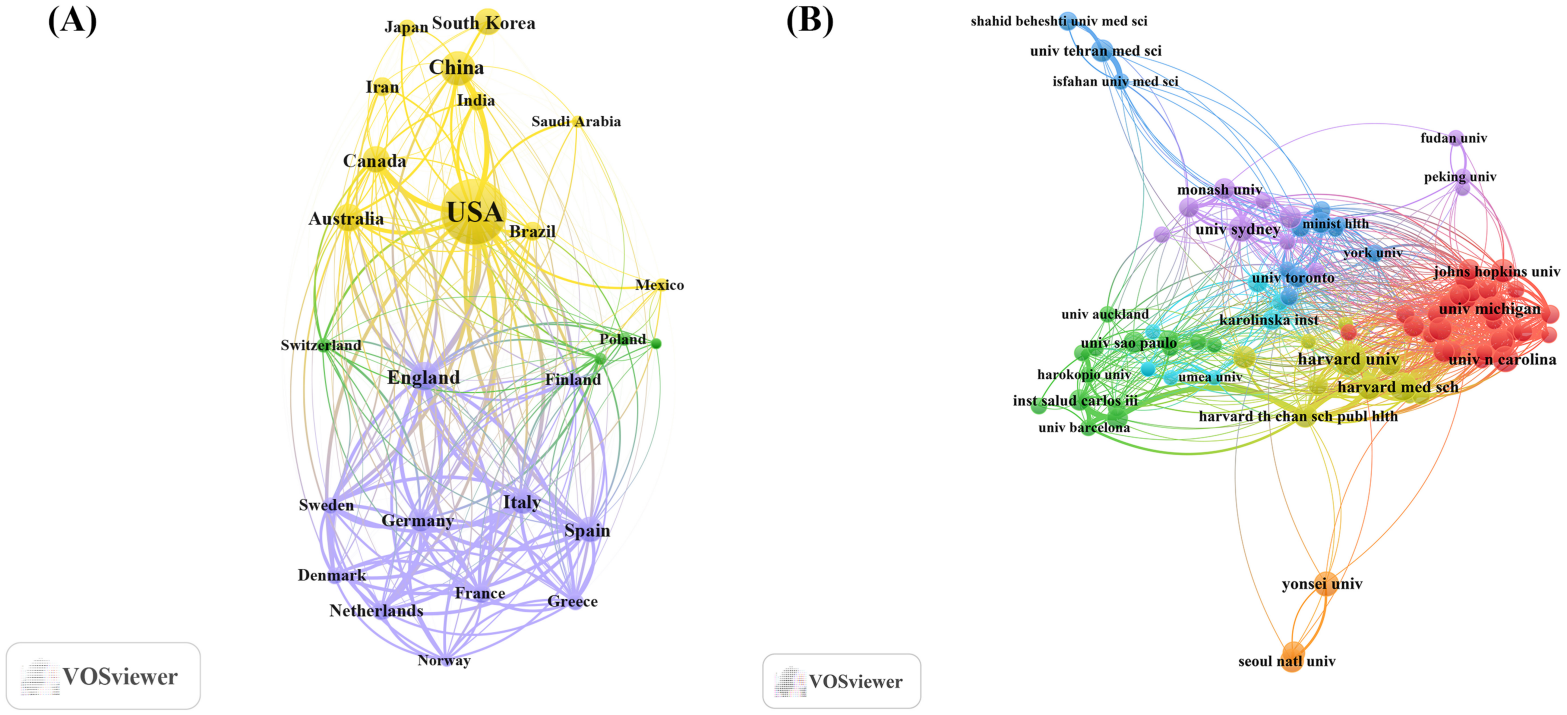
Map of countries/regions and institutions involved in research for exercise and nutrition in diabetes. **(A)** Map of cooperation between different countries. **(B)** Map of cooperation between different institutions.

An analysis of the corresponding authors' countries (Table 1, Figure 1B) indicated that the United States led the field with a remarkable output of 1,431 publications, accounting for 29.9% of all documents, followed by China (*n*= 432), South Korea (*n*= 276), Australia (*n*= 209), and Canada (*n*=193), reflecting active global engagement in this domain. Among the top 10 most relevant countries, South Korea (10.9%), the United States (15.6%), and China (17.8%) reported lower multiple country publications (MCP) ratios, suggesting a stronger emphasis on domestic research output. In contrast, European countries such as UK (46.2%), Germany (34.9%), and Spain (33.1%) exhibited high proportions of MCP, reflecting active cross-border cooperation. Furthermore, the collaboration map (Figure 2A) reinforced this pattern, with well-defined European clusters marked by strong link strengths. These findings underscore the central role of European countries as major collaborative hubs in this research field, highlighting a shared commitment to knowledge exchange and common scientific goals.

**Table 1:** Most relevant countries by corresponding authors of exercise and nutrition in diabetes.

**Country**	**Articles**	**Freq (%)**	**SCP**	**MCP**	**MCP_Ratio (%)**
USA	1,431	29.9	1,208	223	15.6
China	432	9.0	355	77	17.8
South Korea	276	5.8	246	30	10.9
Australia	209	4.4	149	60	28.7
Canada	193	4.0	132	61	31.6
Spain	181	3.8	121	60	33.1
Italy	168	3.5	124	44	26.2
United Kingdom	156	3.3	84	72	46.2
Iran	139	2.9	99	40	28.8
Germany	126	2.6	82	44	34.9

MCP, Multiple country publication; SCP, Single country publication.

As shown in Table 2, institutional contributions to the research on exercise and nutrition in diabetes were highly concentrated in the United States, which accounted for 8 out of the top 10 most productive affiliations. Harvard University ranked first with 520 publications, followed by the University of California System (*n*= 263), and several affiliated institutions including Harvard University Medical Affiliates (*n*= 251), Harvard T.H. Chan School of Public Health (*n*= 175), and Harvard Medical School (*n*= 163). Although international co-authorships were present (24.5%, Bibliometrix), clusters formed within South Korea, Australia, China, and the United States, as visually evident in Figure 2B, indicating that institutional collaborations display a tendency toward regional affinity.

**Table 2:** Top 10 most relevant affiliations of exercise and nutrition in diabetes.

**Affiliation**	**Country**	**Articles**
Harvard University	USA	520
University of California System	USA	263
Harvard University Medical Affiliates	USA	251
Harvard T.H. Chan School of Public Health	USA	175
University of North Carolina	USA	169
Harvard Medical School	USA	163
Johns Hopkins University	USA	161
University of North Carolina Chapel Hill	USA	141
University of London	United Kingdom	137
CIBER - Centro de Investigación Biomédica en Red	Spain	135

### 3.2 Source analysis and visualization

To investigate the scholarly sources contributing most significantly to the field of exercise and nutrition in diabetes research, a multifaceted analysis was conducted. Publication and citation data were derived using the Bibliometrix R package, with visualization via ggplot2, while co-cited journal networks were constructed using VOSviewer. The journal impact factors (IFs) were retrieved from Journal Citation Reports (JCR) in 2025.

**Figure 3:**
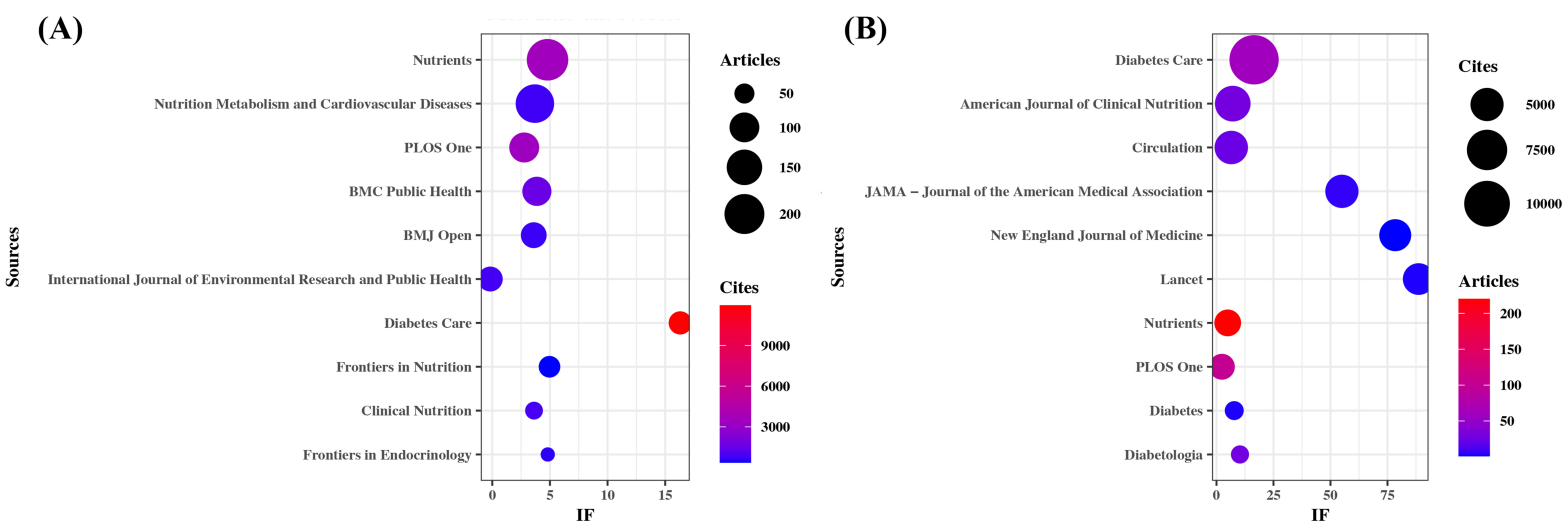
Sources with the largest number of articles published and the sources with the largest number of citations. **(A)** Most relevant sources. **(B)** Most cited sources.

As shown in Table 3 and Figure 3A, *Nutrients* (*n*= 220, IF = 5.0) stood out as the most prolific source, followed by *Nutrition Metabolism and Cardiovascular Diseases* (*n*= 183, IF = 3.7), *PLoS One* (*n*= 101, IF = 2.6), *BMC Public Health* (*n*= 95, IF = 3.6), and *BMJ Open* (*n*= 74, IF = 3.4). Many of these are open-access, multidisciplinary journals, highlighting a commitment to broad accessibility and scientific integration.

**Table 3:** Top 10 most relevant sources of exercise and nutrition in diabetes.

**Sources**	**Articles**	**Cites**	**IF (2025)**
Nutrients	220	3,578	5.0
Nutrition Metabolism and Cardiovascular Diseases	183	778	3.7
PLOS One	101	3,438	2.6
BMC Public Health	95	1,553	3.6
BMJ Open	74	705	3.4
International Journal of Environmental Research and Public Health	69	840	0.0
Diabetes Care	61	11,955	16.6
Frontiers in Nutrition	55	365	5.1
Clinical Nutrition	45	892	3.5
Frontiers in Endocrinology	42	587	4.6

**Figure 4:**
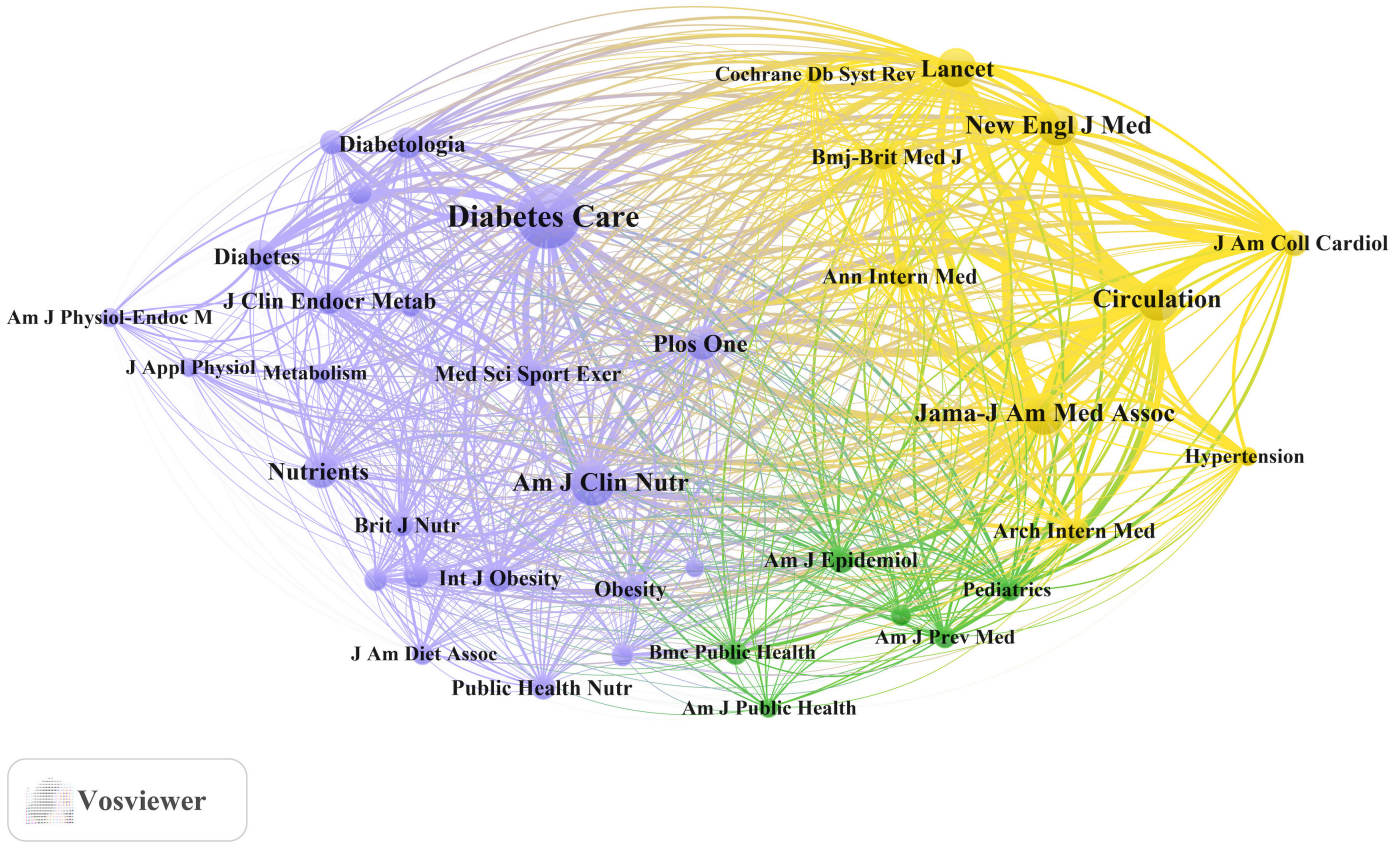
Co-cited journals related to exercise and nutrition in diabetes.

Table 4 and Figure 3B present the most frequently cited sources, including *Diabetes Care* (*n*= 11,955, IF = 16.6), *The American Journal of Clinical Nutrition* (*n*= 5,755, IF = 6.9), Circulation (*n*= 5,066, IF = 6.7), *JAMA – Journal of the American Medical Association* (*n*= 5,022, IF = 55.0), and *The New England Journal of Medicine* (*n*= 4,676, IF = 78.5). Importantly, these journals also emerged as central nodes in the co-cited source network (Figure 4) with the highest total link strengths, collectively underscoring their roles as intellectual hubs in shaping diabetes research. Nevertheless, it is worth noting that studies focusing on exercise and nutrition in diabetes have rarely been published in high-impact general medical journals, indicating a potential disconnect between this subfield and the broader biomedical discourse.

**Table 4:** Top 10 most cited sources of exercise and nutrition in diabetes.

**Sources**	**Cites**	**Articles**	**IF (2025)**
Diabetes Care	11,955	61	16.6
American Journal of Clinical Nutrition	5,755	29	6.9
Circulation	5,066	24	6.7
JAMA - Journal of the American Medical Association	5,022	6	55.0
New England Journal of Medicine	4,676	1	78.5
Lancet	4,590	3	88.5
Nutrients	3,578	220	5.0
PLoS ONE	3,438	101	2.6
Diabetes	2,892	3	7.5
Diabetologia	2,886	28	10.2

### 3.3 Analysis of cited publications and citation bursts

Using the bibliometrix package in R, we identified the top 25 most cited references in the field of exercise and nutrition in diabetes (Table 5). These publications, collectively cited 64,768 times, were sourced from 18 distinct journals. Notably, Circulation contributed the largest number of highly cited articles (*n*= 6), underscoring its central role in disseminating influential medical research. The top three cited references included the 2017 and 2019 editions of the “Heart Disease and Stroke Statistics” update from the American Heart Association (AHA), and the “2019 ACC/AHA Guideline on the Primary Prevention of Cardiovascular Disease.” These works emphasized physical activity and nutrition as core health behaviors, and positioned diabetes as a key factor contributing to cardiovascular risk. The prominence of these guidelines highlighted the sustained efforts by the AHA and the American College of Cardiology (ACC) since the 1980s to translate scientific evidence into clinical practice, providing essential resources for clinicians, researchers, and policymakers committed to improving public health outcomes.

**Table 5:** Top 25 cited references related to exercise and nutrition in diabetes.

**Paper**	**DOI**	**Total citations**	**TC per year**	**Normalized TC**
Benjamin EJ, 2017, CIRCULATION	doi: 10.1161/CIR.0000000000000485	10,792	1,199.11	115.59
Benjamin EJ, 2019, CIRCULATION	doi: 10.1161/CIR.0000000000000659	7723	1,103.29	67.27
Arnett DK, 2019, CIRCULATION	doi: 10.1161/CIR.0000000000000677	5,613	801.86	48.89
Arnett DK, 2019, CIRCULATION-a	doi: 10.1161/CIR.0000000000000678	5,613	801.86	48.89
Piepoli MF, 2016, ATHEROSCLEROSIS	doi: 10.1016/j.atherosclerosis.2016.05.037	3,141	314.10	35.19
Vos T, 2016, LANCET	doi: 10.1016/s0140-6736(16)31678-6	2,868	286.80	32.13
Popkin BM, 2012, NUTR REV	doi: 10.1111/j.1753-4887.2011.00456.x	2,823	201.64	28.36
Rinninella E, 2019, MICROORGANISMS	doi: 10.3390/microorganisms7010014	2,160	308.57	18.81
Poirier P, 2006, CIRCULATION	doi: 10.1161/CIRCULATIONAHA.106.171016	2,122	106.10	18.97
Piepoli MF, 2016, EUR HEART J	doi: 10.1093/eurheartj/ehw106	1,918	191.80	21.49
Hruby A, 2015, PHARMACOECONOMICS	doi: 10.1007/s40273-014-0243-x	1,879	170.82	27.21
Lin JD, 2005, CELL METAB	doi: 10.1016/j.cmet.2005.05.004	1,742	82.95	15.29
Zeevi D, 2015, CELL	doi: 10.1016/j.cell.2015.11.001	1,742	158.36	25.23
Booth FW, 2012, COMPR PHYSIOL	doi: 10.1002/cphy.c110025	1,709	122.07	17.17
Galicia-Garcia U, 2020, INT J MOL SCI	doi: 10.3390/ijms21176275	1,598	266.33	57.66
Mozaffarian D, 2016, CIRCULATION	doi: 10.1161/CIRCULATIONAHA.115.018585	1,512	151.20	16.94
Shai I, 2008, NEW ENGL J MED	doi: 10.1056/NEJMoa0708681	1,427	79.28	12.42
Perk J, 2012, EUR HEART J	doi: 10.1093/eurheartj/ehs092	1,406	100.43	14.13
Vartanian LR, 2007, AM J PUBLIC HEALTH	doi: 10.2105/AJPH.2005.083782	1,303	68.58	12.76
Kushi LH, 2012, CA-CANCER J CLIN	doi: 10.3322/caac.20140	1,002	71.57	10.07
Malik VS, 2013, NAT REV ENDOCRINOL	doi: 10.1038/nrendo.2012.199	995	76.54	17.80
Melamed ML, 2008, ARCH INTERN MED	doi: 10.1001/archinte.168.15.1629	992	55.11	8.63
Giovannucci E, 2008, ARCH INTERN MED	doi: 10.1001/archinte.168.11.1174	923	51.28	8.03
He CC, 2012, NATURE	doi: 10.1038/nature10758	913	65.21	9.17
Kyu HH, 2016, BMJ-BRIT MED J	doi: 10.1136/bmj.i3857	852	85.20	9.55

**Figure 5:**
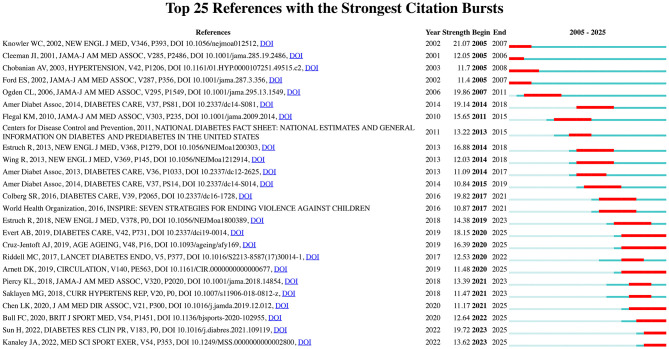
Top 25 references with the strongest citation bursts in exercise and nutrition in diabetes.

To identify the most significant citation bursts related to exercise and nutrition in diabetes, we utilized CiteSpace (selection criteria: top 25; number of states: 2; minimum duration: 2). This analysis yielded 249 references with the strongest citation bursts, with the top 25 displayed in Figure 5. Among these, the three citations exhibiting the most pronounced bursts were: (1) “Reduction in the Incidence of Type 2 Diabetes with Lifestyle Intervention or Metformin” (Strength: 21.07); (2) “Prevalence of Overweight and Obesity in the United States, 1999–2004” (Strength: 19.86); and (3) “Physical Activity/Exercise and Diabetes: A Position Statement of the American Diabetes Association” (Strength: 19.82).

Furthermore, the three most cutting-edge citation bursts were associated with the following references: (1) “World Health Organization 2020 Guidelines on Physical Activity and Sedentary Behavior;” (2) “IDF Diabetes Atlas: Global, Regional and Country-Level Diabetes Prevalence Estimates for 2021 and Projections for 2045;” and (3) “Exercise/Physical Activity in Individuals with Type 2 Diabetes: A Consensus Statement from the American College of Sports Medicine.”

Collectively, the co-citation and burst detection analyses highlighted two focal points within the research landscape of exercise and nutrition in diabetes: (1) Lifestyle interventions in diabetes prevention and management, highlighting the pivotal role of diet and physical activity in the primary and secondary prevention of diabetes and related cardiovascular diseases; (2) Epidemiological trends in diabetes, reflecting the global rise in prevalence, incidence, and disability burden, and informing policy responses through data-driven insights.

### 3.4 Keyword clusters and evolution of themes

For the keyword co-occurrence analysis, the minimum number of occurrences of a keyword was set at 50. Keywords such as “nutrition,” “exercise,” “physical activity,” and “diabetes” were excluded because their high frequency resulting from presence in the search strategy could bias the analysis and overshadow other relevant keywords.

keywords play a key role in summarizing core content and mapping the knowledge structure of a field. Table 6 presents the top 10 most frequently occurring keywords, highlighting the primary research focuses within the dataset. The most frequent keywords were “obesity” (*n*= 1,156), “risk” (*n*= 833), “type 2 diabetes” (*n*= 720), “prevalence” (*n*= 660), and “cardiovascular diseases” (*n* = 611).

**Table 6:** Top 10 frequent keywords related to exercise and nutrition in diabetes.

**Rank**	**Keywords**	**Count**
1	Obesity	1,156
2	Risk	833
3	Type 2 diabetes	720
4	Prevalence	660
5	Cardiovascular diseases	611
6	Metabolic syndrome	600
7	Insulin resistance	587
8	Risk factors	545
9	Health	526
10	Associations	497

**Figure 6:**
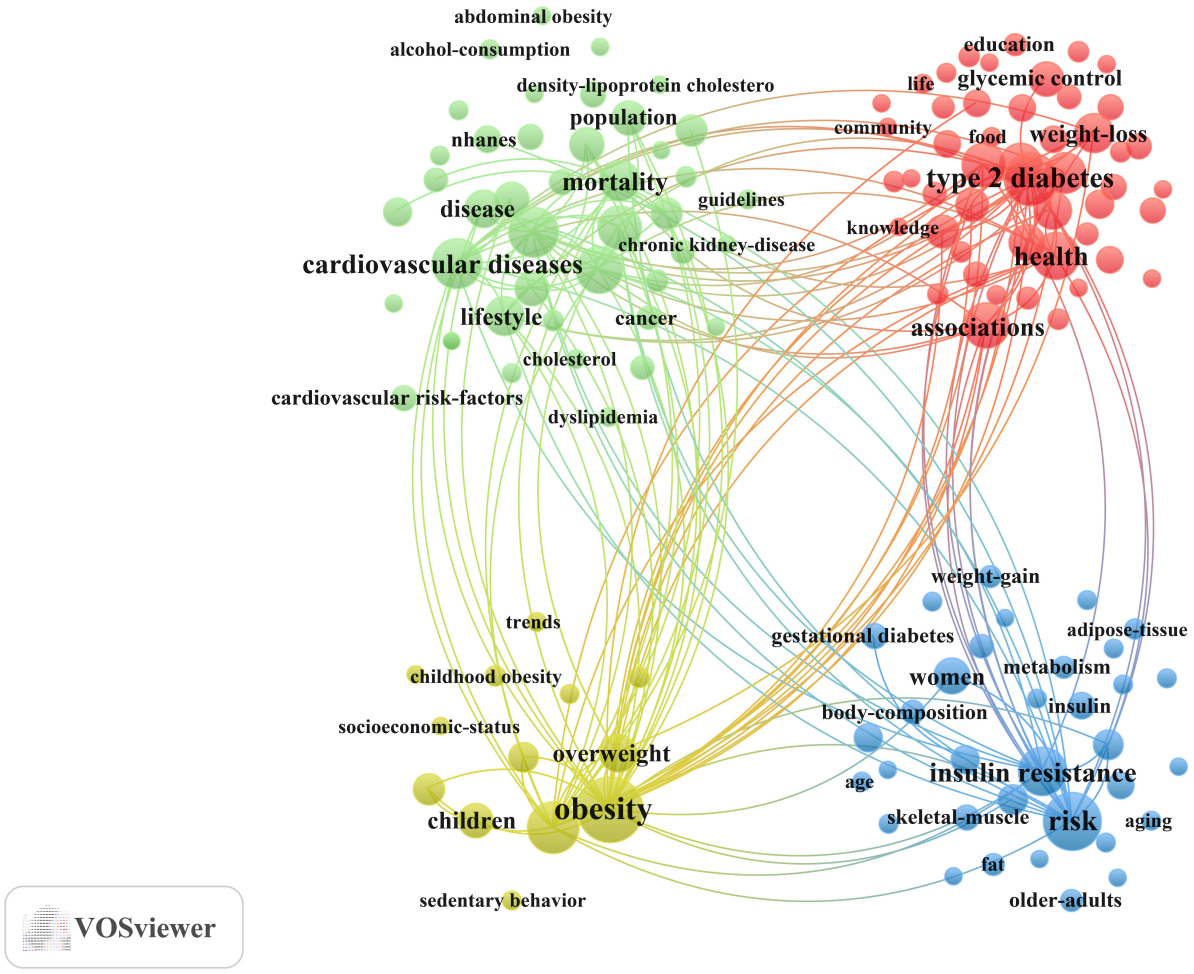
Keyword co-occurrence map of publications on exercise and nutrition in diabetes.

Subsequently, different keyword variations were merged, and a total of 138 keywords with a minimum occurrence of 50 were selected to construct the keyword clustering map (Figure 6). Cluster analysis revealed four distinct clusters: (1) Management of diabetes-related complications through exercise and dietary approaches (Green cluster), comprising 42 keywords including cardiovascular diseases, metabolic syndrome, coronary-heart-disease, hypertension, myocardial-infarction, chronic kidney-disease, all-cause mortality, dietary patterns, lifestyle, guidelines, among others; (2) Lifestyle interventions in adults with prediabetes and diabetes (Red cluster), comprising 49 keywords including associations, diabetes prevention, self-management, lifestyle intervention, glycemic control, mediterranean diet, food, impact, behaviors, adherence, medical nutrition therapy, among others; (3) Physical activity and nutritional management in children and adolescents with early-life risk of diabetes (Yellow cluster), comprising 13 keywords including children, childhood obesity, overweight, nutrition transition, sedentary behavior, adiposity, among others; (4) Exercise and nutritional strategies in women and aging populations for metabolic health (Blue cluster), comprising 34 keywords including women, pregnancy, gestational diabetes, older-adults, insulin resistance, hyperglycemia, metabolism, protein, supplementation, skeletal-muscle, sarcopenia, among others. A complete list of the keywords included in the four clusters is provided in Supplementary material 2.

Additionally, keyword clustering was conducted using the Scopus dataset. After merging synonyms and setting the minimum keyword occurrence at 300, 169 keywords were identified by VOSviewer, forming three clusters (Supplementary Figure 2). The first cluster focuses on evidence-based exercise and nutrition practice in defined populations with and at risk of diabetes. It draws upon various study designs, including prospective studies, major clinical trials, cohort analyses, and cross-sectional studies, to investigate interventions across diverse demographic groups such as females, males, adults, young adults, and older adults. The second cluster centers on exercise and dietary modifications in diabetes and related complications management, covering a broad range of keywords related to diet therapy, diet supplementation, lifestyle modification, complication, comorbidly, sedentary lifestyle, practice guideline, and health promotion. Both of these thematic areas are consistent with the findings from the WoSCC analysis. In contrast, the third cluster (Blue), which emerged more prominently in the Scopus dataset, focuses on assessing exercise and dietary interventions in diabetes through comparative studies using biomarker and anthropometric indicators. It includes clinical measures such as c reactive protein, creatinine, systolic blood pressure, diastolic blood pressure, cholesterol blood level, and triacylglycerol blood level. All keywords included in the three clusters are available in Supplementary material 3.

**Figure 7:**
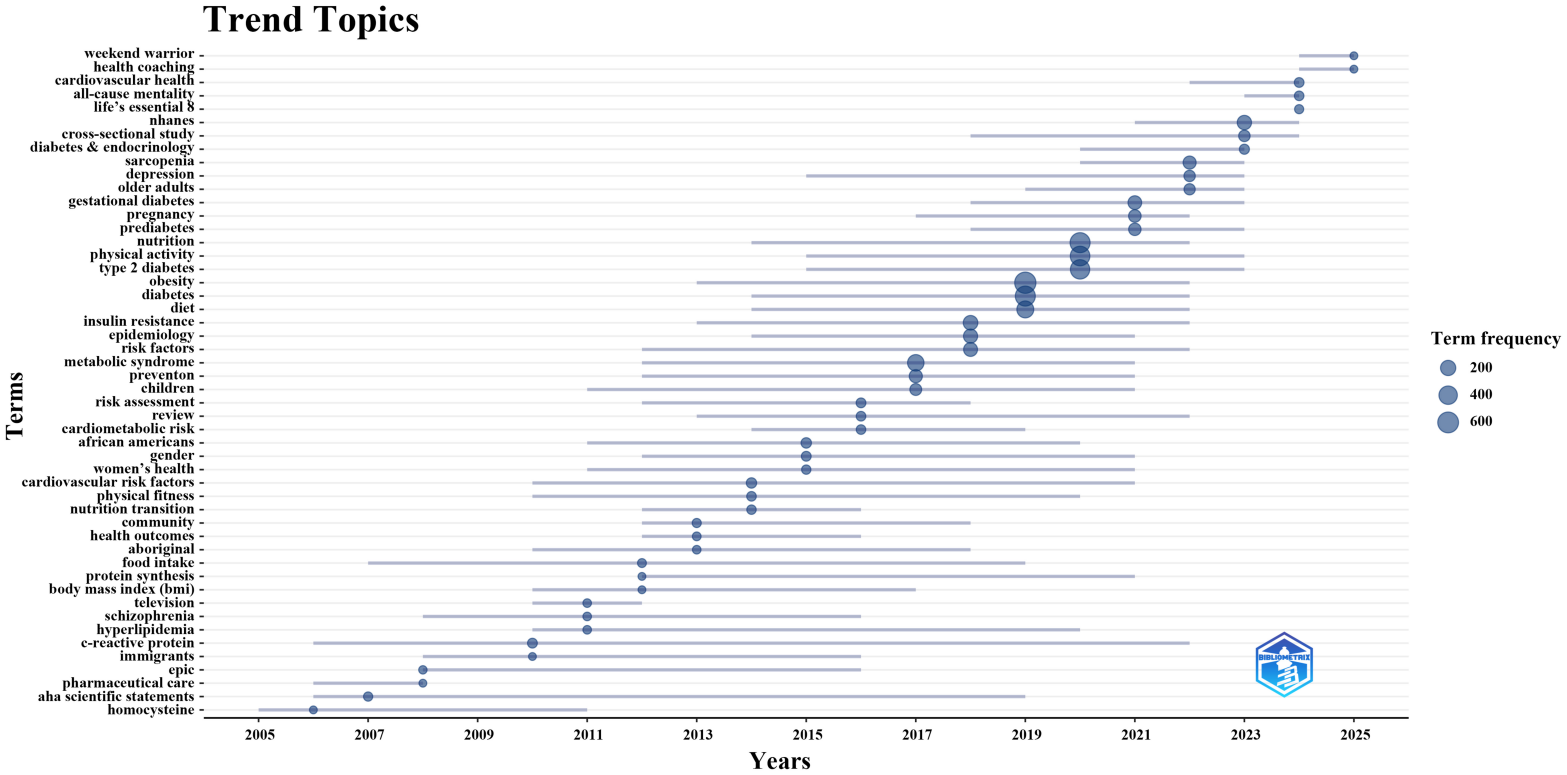
Trend topics on exercise and nutrition in diabetes.

To further investigate the evolving trend topics, we employed the Bibliometrix package in the R programming environment to construct a dynamic thematic evolution map (Figure 7). Between 2005 and 2010, research focused on metabolic biomarkers (e.g., homocysteine, C-reactive protein) and pharmaceutical care, reflecting the integration of early mechanistic insights with clinical guidance in diabetes management. From 2011 to 2014, increasing attention was given to lifestyle factors, including physical fitness and the nutrition transition. Subsequently, during 2015–2022, topics such as obesity, physical activity, and nutrition dominated the field, with exercise and nutrition interventions for diabetes becoming a central theme. This period also witnessed a shift toward more population-specific research, addressing subgroups such as children, pregnant women, older adults, and exploring sex and ethnic disparities in health. Since 2023, studies have increasingly incorporated large-scale data approaches, such as the National Health and Nutrition Examination Survey (NHANES) and cross-sectional study designs. At the same time, emerging topics such as Life's Essential 8, health coaching, and Weekend Warrior reflect a growing emphasis on evidence-based, person-centered, and innovative approaches to positive lifelong health promotion.

## 4 Discussion

### 4.1 General information

To better capture the research hotspots and trends in exercise and nutrition in diabetes, we conducted a bibliometric analysis and data visualization based on 4,793 unique publications from the WoSCC and 9,187 from Scopus, covering the period from January 1, 2005, to July 3, 2025. The results reveal a steady increase in publication volume from 2005 to 2024, reflecting a growing research interest in lifestyle-based strategies for the prevention and management of diabetes. Notably, both databases demonstrate a marked surge in publications between 2019 and 2021. This sharp increase may be attributed to the release of multiple high-impact guidelines and consensus statements issued in 2019 by major health organizations, including ADA, AHA, ACC, ACSM, and WHO, which collectively emphasized the pivotal role of exercise and nutrition in addressing diabetes, prediabetes, and cardiovascular disease risk. Importantly, these influential documents not only rank among the most cited references but also exhibit strong citation bursts, underscoring their profound impact on shaping subsequent research directions. Furthermore, research activity remains high in 2025, indicating sustained academic attention and ongoing engagement with this evolving field.

Among the countries contributing to this field, the United States leads with 1,431 publications (29.9%), with its institutions occupying 8 of the top 10 most productive affiliations, featuring Harvard University and its affiliates at the forefront. The strong research output may be linked to substantial public investment, with the National Institutes of Health (NIH) allocating over $1 billion annually to diabetes research, alongside increased support to strengthen the research workforce through training grants, fellowships, and career development awards (36). In the source analysis, Nutrients emerges as the most prolific journal, followed by Nutrition Metabolism and Cardiovascular Diseases and PLOS One. Diabetes Care stands out among the most frequently cited sources, alongside several other high-impact journals. Looking forward, future efforts could aim to further align research in exercise and nutrition in diabetes with top-tier medical outlets to elevate its academic and translational impact.

### 4.2 Hotspots and development trends

Building on the analyses of co-citation, burst detection, keyword frequency, keyword clustering, and thematic evolution, we identified potential research hotspots regarding the exercise and nutrition in diabetes, mainly focusing on three aspects. First, research has focused on intervention approaches, examining the underlying mechanisms, therapeutic efficacy, and safety profiles of various exercise types, nutritional strategies, and their combined applications in both T1D and T2D. Second, research has concentrated on managing diabetes-related complications through exercise and nutrition, highlighting metabolic benefits, clinical recommendations, and contraindications across various complications. Third, studies have targeted population-specific exercise and nutrition interventions for older adults, children, and women with gestational diabetes, highlighting evidence-based guidelines and varied strategies to enhance adherence.

#### 4.2.1 Exercise, nutrition, and combined interventions in diabetes, addressing mechanistic insights, therapeutic efficacy, and risk mitigation

Aerobic exercise (e.g., walking, dancing, and swimming), which derives energy from the oxidative breakdown of glycogen and fat, can rapidly improve blood glucose levels and insulin sensitivity when performed at moderate intensity. In contrast, Anaerobic exercise (e.g., weightlifting, sprinting, and planking) relies on the phosphagen and lactic acid systems for energy supply. Resistance training, as a key anaerobic modality, can increase muscle mass and thereby facilitate glucose uptake (37). Notably, combining exercise types, such as in high-intensity interval training (HIIT), have shown superior benefits for glucose metabolism compared to continuous aerobic training (38, 39). For high-risk adults of T2D, exercise effectively contributes to prevention and delay of disease onset, with at least 150 minutes of moderate to vigorous physical activity per week recommended (40). Nonetheless, for T1D, managing diverse physical activities poses significant challenges to patients and clinicians alike (41). Both aerobic and anaerobic exercise can cause delayed-onset hypoglycemia during recovery, with HIIT potentially posing a greater risk of nocturnal hypoglycemia than aerobic exercise (42). Therefore, in addition to continuous glucose monitoring, personalized insulin and nutritional strategies are crucial. For example, consuming 60 grams or more of carbohydrates per hour during aerobic exercise may reduce hypoglycemia risk (41).

In addition, medical nutrition therapy (MNT) delivered by registered dietitian nutritionists (RDNs) is strongly supported by evidence for improving glycemic control, with absolute A1C reductions of up to 2.0% in T2D and up to 1.9% in T1D over 3–6 months (43). In T2D and prediabetes, dietary patterns such as the Mediterranean, low-fat, or low-carbohydrate diets are effective for glycemic control and disease prevention (44, 45). For T1D, very low-carbohydrate (VLC) diets may confer potential benefits, though large, long-term clinical trials are needed to substantiate these findings (46). Pending stronger evidence for specific patterns, nutrition strategies should prioritize non-starchy vegetables, minimize added sugars and refined grains, and favor whole foods over ultra-processed products (19). Furthermore, no universal macronutrient distribution is ideal; instead, it should be individualized based on metabolic goals, physical activity, preferences, and food access, with total energy intake supporting weight management (47). Regarding micronutrients, routine supplementation is not supported unless deficiency is present (48). However, multivitamin use may be appropriate in certain populations, including women planning pregnancy, older adults, vegetarians, or those on macronutrient-restricted diets (49).

Under the widely accepted consensus on nutrition and exercise interventions in diabetes management, their complex interplay and cumulative effects, particular the influence on key metabolic organs such as skeletal muscle and adipose tissue, have garnered increasing attention (50). Skeletal muscle protein breakdown induced by nutritional imbalance leads to muscle loss and reduced metabolic rate, whereas a balanced diet and exercise, especially resistance training, can effectively mitigate these effects (51). Adipose tissue metabolism is responsive to both dietary and exercise interventions, and their combination can enhance metabolic flexibility and promote synergistic metabolic adaptations (52). As demonstrated by a 16-week intervention, combined diet and exercise led to superior improvements in clinical outcomes among individuals with T2D, likely mediated by improvements in hepatic insulin sensitivity and β-cell function from dietary changes, as well as enhanced peripheral insulin sensitivity resulting from exercise (53). Additionally, endurance training in both fasted and fed states has been shown to be safe and effective, with postprandial exercise appearing more beneficial for HbA1c control (54). In adults with T1D, nutrition around exercise must be carefully managed, considering individualized macronutrient needs, fluid intake, supplementation, and if necessary, insulin dosing adjustments to prevent hypoglycemia, optimize performance, and support recovery (55). Notably, fasted morning aerobic activity is gaining popularity for its lower hypoglycemia risk, while intense pre-breakfast exercise may elevate glucose depending on intensity and duration (56, 57).

#### 4.2.2 Exercise and nutrition in managing long-term complications of diabetes, highlighting metabolic benefits, clinical recommendations, and contraindications

Diabetes significantly accelerates atherosclerosis, posing a major risk for cardiovascular disease (CVD) and peripheral artery disease (PAD) (58). Both aerobic and resistance training can improve endothelial function and cardiovascular health, with supervised programs benefiting even those with established CVD (59, 60). In PAD, moderate-intensity walking, arm ergometry, and cycling are also beneficial (61). Nutritionally, low-carbohydrate diets outperform low-fat diets in glycemic and CVD risk control, while replacing saturated fats with unsaturated fats reduces LDL-C and total cholesterol (19). Diabetic kidney disease (DKD), affecting approximately 30% of individuals with diabetes, is a leading cause of mortality (62). Physical activity can enhance functional capacity and quality of life across DKD stages, including during dialysis (58). Dietary management should be individualized, with attention to potassium, phosphate, sodium, and energy intake. Routine protein restriction is not recommended; however, in cases of marked proteinuria, soy-based protein sources may offer additional cardiovascular benefits (19, 63).

Additionally, diabetic neuropathy (DN) is a common microvascular complication, present in nearly 50% of individuals with diabetes (43). Diabetic retinopathy remains a leading cause of blindness in working-age populations; while low- to moderate-intensity exercise may confer some benefits, vigorous aerobic or resistance training is contraindicated in cases of proliferative or severe non-proliferative retinopathy due to risks of vitreous hemorrhage or retinal detachment (64, 65). Peripheral neuropathy affects up to 40% of diabetes patients, particularly the lower limbs, and mild-to-moderate physical activity may help delay onset (64). However, individuals with foot ulcers or injuries should limit activity to non-weight-bearing exercises (58). Cardiac autonomic neuropathy (CAN), linked to heart rate abnormalities, silent myocardial infarction, and increased mortality, has been shown to benefit from moderate-intensity aerobic training, though pre-exercise screening and physician clearance are recommended (66). From a nutritional perspective, vitamin B12 deficiency, linked to peripheral and autonomic neuropathy, is common and warrants supplementation (67). Dietary antioxidants such as alpha-lipoic acid and compounds like nicotinamide riboside may offer neuroprotective effects (68). Translational research also implicates high-fat diets in the progression of DN, suggesting dietary modulation as a potential intervention strategy (69).

#### 4.2.3 Population-specific exercise and nutrition strategies for older adults, children, and women with and at risk of diabetes, incorporating evidence-based approaches and adherence guidance

Beyond general strategies for adults, a growing body of research has emphasized tailored exercise and nutrition interventions for specific populations, notably older adults and children. Aging is associated with significant alterations in body composition, including reduced lean muscle mass and increased adiposity, particularly visceral fat, which collectively elevate the risk of diabetes (70). Aerobic exercise has long been recommended to improve glucose tolerance and prevent diabetes, while also benefiting common comorbidities in older adults such as hypertension, CVD, and osteoporosis (71). In recent years, resistance training has gained recognition as an essential component of fitness regimens among the elderly, enhancing muscular strength, promoting spontaneous physical activity, and increasing nutritional demands (72, 73). From a nutritional standpoint, adequate intake of high-quality protein (1.0–1.5 g/kg/day), vitamin D, and acid–base balance diet are crucial for maintaining metabolic and musculoskeletal health (74, 75). Notably, when combined with resistance training, protein supplementation may synergistically promote greater improvements in muscle mass and strength among frail older individuals (76, 77). Moreover, to minimize risks and enhance adherence in older adults, initial health screenings and structured warm-up and cool-down routines should be prioritized, with community-based strategies encouraging lifestyle change (70).

The global rise in childhood overweight and obesity, projected to reach 30.0% by 2030, has significantly increased the risk of diabetes in youth (78). Children and young adults with a family history of diabetes also represent a particularly high-risk group, with findings from national surveys in the United States, Malaysia, and Korea showing increased likelihood of developing T2D and metabolic disorders, often accompanied by modifiable lifestyle risk factors (79–81). Extensive evidence supports that regular physical activity can significantly reduce metabolic risk in children (82). For instance, 13 weeks of daily aerobic exercise (20–40 min) led to improved insulin resistance, reduced adiposity, and enhanced fitness in overweight children across sexes and ethnicities (83). Nutritionally, a balanced diet rich in vitamins and minerals, whole grains, dairy products, fruits, and vegetables, while limiting the intake of high-sugar, high-fat, and high-salt foods, is essential for healthy growth and glycemic control in children (84). In addition, establishing healthy eating patterns with family support, such as regular mealtimes, family meals, and avoiding screen-time eating, can further improve children's nutritional status (85). Moreover, combined diet and exercise interventions have been shown to reduce diabetes risk by up to 60% in youth with impaired glucose tolerance and lower the need for medication in those with T2D (86). To ensure the safety and efficacy of exercise and nutrition strategies, it is crucial to implement glucose monitoring, individualized nutritional adjustments, and education efforts engaging families, teachers, and peers (87). Notably, family history represents a key criterion for identifying individuals who should be prioritized for intensive lifestyle modification counseling by health care providers (79).

Additionally, increasing attention has been paid to women, particularly during pregnancy, where gestational diabetes (GD) poses specific challenges and opportunities for intervention to support both maternal and fetal health (88). A secondary analysis revealed that women with GD had a 71% higher risk of developing T2D within three years; however, this risk could be reduced by approximately 50% through lifestyle interventions (89). Nutritionally, restrictive diets that severely limit specific macronutrients should be avoided in GD (90). Instead, balanced diets emphasizing whole foods (e.g., fruits, vegetables, legumes, whole grains, lean proteins, and omega-3 fatty acids) are recommended to promote healthy weight (91). Moreover, micronutrient-based supplements like probiotics and inositol may offer convenient and cost-effective support (92). Evidence from eight randomized controlled trials indicates that combining diet and exercise interventions leads to superior glycemic outcomes in GD, reducing both fasting and postprandial glucose levels (93). Guidelines recommend at least 150 min of moderate-intensity aerobic exercise per week, with some studies endorsing the inclusion of resistance training (94, 95). Ensuring safety during pregnancy is paramount; thus, high-fall-risk sports, supine exercises after the first trimester, prolonged standing, and scuba diving should be avoided, with exercise programs individualized by professionals (96).

### 4.3 Limitations and considerations

This study offers a systematic and replicable bibliometric approach, serving as a reliable reference for future investigations. However, several limitations should be acknowledged. First, the analysis was restricted to publications indexed in WoSCC and Scopus. While these sources are considered authoritative and reliable, this restriction may have led to the omission of relevant studies from other databases. Second, only English-language publications from the past two decades were considered, potentially excluding valuable non-English research and limiting the generalizability of the findings. Third, only documents classified as Articles or Reviews were considered, ensuring data quality and consistency but leaving out other publication types that may contain significant evidence. Together, these restrictions in data collection may have contributed to selection bias by overlooking certain relevant studies. Fourth, the analysis focused on publication quantity and citation metrics without assessing the quality of individual studies; all included publications were weighted equally, which may obscure differences in research impact or validity. Fifth, citation bias is an inherent limitation of bibliometric analyses, as highly cited studies may disproportionately influence networks and thematic trends, potentially exaggerating the prominence of certain regions or topics. Finally, regional disparities in research capacity could have affected publication outputs and co-authorship networks, resulting in uneven representation across countries. Despite these limitations, the combined use of WoSCC and Scopus, along with complementary bibliometric tools, helps mitigate some of these biases and supports the robustness of the overall findings.

### 4.4 Implications for future research and practice

This bibliometric analysis highlights several emerging directions with important implications for future research and practice in exercise and nutrition for diabetes management. First, there is a growing shift from a disease-centric approach toward a life course–oriented paradigm that emphasizes sustained health promotion and risk prevention across all stages of life (97). Pregnancy and the surrounding periods (preconception and postpartum) are particularly critical for shaping maternal health trajectories and intergenerational outcomes, especially in Australia's Northern Territory, where diabetes disproportionately affects Aboriginal and Torres Strait Islander women (98, 99). Therefore, the DIABETES Across the LIFECOURSE project applied a co-design approach to engage women, families, communities, and health services in identifying priorities and strategies. Key focuses included improving access to nutritious foods and opportunities for physical activity, strengthening cultural connections, enhancing communication about diabetes risks, and addressing barriers that prevent women from prioritizing their own health. By enabling collaborative decision-making, this approach supported self-determination and enhanced the acceptability and lasting benefits of lifestyle interventions aimed at reducing diabetes risk (100).

Second, an evolving focus centers on one-on-one health coaching beyond traditional healthcare settings. Personalized and interactive support, delivered by peers or professionals, has been shown to empower patients by enhancing self-efficacy, accountability, and clinical outcomes, making it essential for effective diabetes self-management (101). However, traditional face-to-face coaching poses challenges in scalability, partly due to its reliance on intensive personal contact and the associated burden on healthcare resources. To address these limitations, recent advances have explored technology-assisted health coaching. Digital diabetes prevention programs, delivered via internet platforms, mobile applications, or text messaging, enable scalable information delivery, lifestyle coaching, and peer support (102). eHealth interventions that integrate behavior change techniques (BCTs) and dynamic tailoring strategies show promise in supporting sustainable lifestyle change (103). Looking ahead, further refinement of digital health coaching and broader evaluation of its impact on lifestyle behaviors will be essential to optimize effectiveness and scalability (103, 104).

Third, emerging patterns point to the need to re-evaluate conventional recommendations in favor of more adaptable formats. With rapid urbanization and extended working hours, opportunities for regular exercise during the workweek are limited. In this context, flexible approaches such as the “Weekend Warrior” pattern, characterized by performing all weekly exercise in one or two days, may provide comparable health benefits while better accommodating individuals with time constraints and busy lifestyles (105). Cross-sectional analysis of NHANES 2007–2016 data showed that this pattern correlated with a lower prevalence of diabetes compared with inactivity, with effects similar to those observed in regularly active individuals (106). Further, a prospective NHANES 2007–2018 cohort study among adults with T2D found that adopting this approach was associated with a 40%–50% reduction in all-cause mortality, again comparable to regular activity. Importantly, this strategy may be particularly relevant for adults with diabetes who experience muscle weakness or exercise intolerance that limits weekday activity, and it can be implemented through a combination of leisure-time and occupational activities (107). Recognizing and incorporating such flexible approaches could improve adherence and expand the reach of lifestyle interventions (108).

## 5 Conclusion

Our bibliometric analysis provides a comprehensive overview of the evolving landscape of research on exercise and nutrition in diabetes, highlighting key knowledge domains and emerging trends:

(A) Global contributions: The research landscape features active global participation, with the United States at the forefront of productivity and institutional influence. China, South Korea, Australia, and Canada also play important roles. Notably, Harvard University and its affiliates are prominent institutional contributors in this field.

(B) Core journals: Nutrients has emerged as the most prolific journal in terms of publication volume, while Diabetes Care is the most frequently cited source. Future research should focus on publishing in high-impact medical journals to elevate the field's academic and translational impact.

(C) Intervention approaches: Mechanistic insights, therapeutic efficacy, and risk mitigation of different exercise types, nutritional approaches, and their combined interventions in diabetes have emerged as prominent areas of research interest.

(D) Complication management: The role of exercise and nutrition in managing chronic complications of diabetes, including CVD, PAD, DKD, and DN, has emerged as a major research focus, with particular emphasis on metabolic benefits, clinical recommendations, and contraindications.

(E) Population-specific interventions: Tailored exercise and nutrition strategies for older adults, children, and women with diabetes have emerged as key areas of research, emphasizing evidence-based recommendations and diverse strategies to improve compliance.

(F) Research frontiers: In recent years, cutting-edge strategies have marked a paradigm shift toward life course orientation, individualized self-management support, and novel adaptive interventions for proactive diabetes care.

In summary, our study reveals the strategic trajectory of exercise and nutrition research in diabetes, offering valuable insights into its scientific foundation and translational potential. These findings provide a roadmap for future investigations aimed at optimizing lifestyle interventions and enhancing personalized care strategies in diabetes prevention, management, and positive health promotion.

## Data Availability

The raw data supporting the conclusions of this article will be made available by the authors, without undue reservation.

## References

[1] Association AD. Diagnosis and classification of diabetes mellitus. Diabetes Care. (2014) 37:S81–90. 10.2337/dc14-S081 24357215

[2] Solis-Herrera C Triplitt C Reasner C DeFronzo RA Cersosimo E. Classification of diabetes mellitus. Endotext. (2015).

[3] Delaney MF Zisman A Kettyle WM. Diabetic ketoacidosis and hyperglycemic hyperosmolar nonketotic syndrome. Endocrinol Metab Clin North Am. (2000) 29:683–705. 10.1016/S0889-8529(05)70159-6 11149157

[4] Dhatariya KK Glaser NS Codner E Umpierrez GE. Diabetic ketoacidosis. Nat Rev Dis Primers. (2020) 6:40. 10.1038/s41572-020-0165-1 32409703

[5] Cade WT. Diabetes-related microvascular and macrovascular diseases in the physical therapy setting. Phys Ther. (2008) 88:1322–35. 10.2522/ptj.20080008 18801863 PMC2579903

[6] Bailes BK. Diabetes mellitus and its chronic complications. AORN J. (2002) 76:265–82. 10.1016/S0001-2092(06)61065-X 12194653

[7] Lotfy M Adeghate J Kalasz H Singh J Adeghate E. Chronic complications of diabetes mellitus: a mini review. Curr Diabetes Rev. (2017) 13:3–10. 10.2174/1573399812666151016101622 26472574

[8] Federation ID. IDF Diabetes Atlas 11th Edition (2025).

[9] Saeedi P Petersohn I Salpea P Malanda B Karuranga S Unwin N . Global and regional diabetes prevalence estimates for 2019 and projections for 2030 and 2045: results from the International Diabetes Federation Diabetes Atlas. Diabetes Res Clin Pract. (2019) 157:107843. 10.1016/j.diabres.2019.107843 31518657

[10] Nyenwe EA Jerkins TW Umpierrez GE Kitabchi AE. Management of type 2 diabetes: evolving strategies for the treatment of patients with type 2 diabetes. Metabolism. (2011) 60:1–23. 10.1016/j.metabol.2010.09.010 21134520 PMC3746516

[11] Stevens JW Khunti K Harvey R Johnson M Preston L Woods HB . Preventing the progression to type 2 diabetes mellitus in adults at high risk: a systematic review and network meta-analysis of lifestyle, pharmacological and surgical interventions. Diabetes Res Clin Pract. (2015) 107:320–31. 10.1016/j.diabres.2015.01.027 25638454

[12] Bergenstal RM Bailey CJ Kendall DM. Type 2 diabetes: assessing the relative risks and benefits of glucose-lowering medications. Am J Med. (2010) 123:374.e9–374.e18. 10.1016/j.amjmed.2009.07.017 20362759

[13] Inzucchi SE Bergenstal RM Buse JB Diamant M Ferrannini E Nauck M . Management of hyperglycemia in type 2 diabetes: a patient-centered approach: position statement of the American Diabetes Association (ADA) and the European Association for the Study of Diabetes (EASD). Diabetes Spectrum. (2012) 25:154–71. 10.2337/diaspect.25.3.154 22526604

[14] Nelson KM Reiber G Boyko EJ. Diet and exercise among adults with type 2 diabetes: findings from the third national health and nutrition examination survey (NHANES III). Diabetes Care. (2002) 25:1722–8. 10.2337/diacare.25.10.1722 12351468

[15] Stephenson EJ Smiles W Hawley JA. The relationship between exercise, nutrition and type 2 diabetes. Med Sport Sci. (2014) 60:1–10. 10.1159/000357331 25226796

[16] Colberg SR Sigal RJ Yardley JE Riddell MC Dunstan DW Dempsey PC . Physical activity/exercise and diabetes: a position statement of the American Diabetes Association. Diabetes Care. (2016) 39:2065. 10.2337/dc16-1728 27926890 PMC6908414

[17] Minnock D Annibalini G Le Roux CW Contarelli S Krause M Saltarelli R . Effects of acute aerobic, resistance and combined exercises on 24-h glucose variability and skeletal muscle signalling responses in type 1 diabetics. Eur J Appl Physiol. (2020) 120:2677–91. 10.1007/s00421-020-04491-6 32909059

[18] Association AD. Diabetes mellitus and exercise. Diabetes Care. (2002) 25:s64. 10.2337/diacare.25.2007.S64

[19] Evert AB Dennison M Gardner CD Garvey WT Lau KHK MacLeod J . Nutrition therapy for adults with diabetes or prediabetes: a consensus report. Diabetes Care. (2019) 42:731. 10.2337/dci19-0014 31000505 PMC7011201

[20] Pongrac Barlovic D Harjutsalo V Groop PH. Exercise and nutrition in type 1 diabetes: insights from the FinnDiane cohort. Front Endocrinol. (2022) 13:1064185. 10.3389/fendo.2022.1064185 36619534 PMC9813408

[21] Armstrong D Hanft J Driver V Smith A Lazaro-Martinez J Reyzelman A . Effect of oral nutritional supplementation on wound healing in diabetic foot ulcers: a prospective randomized controlled trial. Diabetic Med. (2014) 31:1069–77. 10.1111/dme.12509 24867069 PMC4232867

[22] Bartlett HE Eperjesi F. Nutritional supplementation for type 2 diabetes: a systematic review. Ophthal Physiol Optics. (2008) 28:503–23. 10.1111/j.1475-1313.2008.00595.x 19076553

[23] Slentz CA Bateman LA Willis LH Granville EO Piner LW Samsa GP . Effects of exercise training alone vs. a combined exercise and nutritional lifestyle intervention on glucose homeostasis in prediabetic individuals: a randomised controlled trial. Diabetologia. (2016) 59:2088–98. 10.1007/s00125-016-4051-z 27421729 PMC5026926

[24] Abdelhafiz AH Sinclair AJ. Diabetes, nutrition, and exercise. Clin Geriatr Med. (2015) 31:439–51. 10.1016/j.cger.2015.04.011 26195102

[25] Magkos F Hjorth MF Astrup A. Diet and exercise in the prevention and treatment of type 2 diabetes mellitus. Nat Rev Endocrinol. (2020) 16:545–55. 10.1038/s41574-020-0381-5 32690918

[26] Kullmann S Valenta V Wagner R Tschritter O Machann J Häring HU . Brain insulin sensitivity is linked to adiposity and body fat distribution. Nat Commun. (2020) 11:1841. 10.1038/s41467-020-15686-y 32296068 PMC7160151

[27] Donthu N Kumar S Mukherjee D Pandey N Lim WM. How to conduct a bibliometric analysis: an overview and guidelines. J Bus Res. (2021) 133:285–96. 10.1016/j.jbusres.2021.04.070

[28] Kumar M George RJ. PS A Bibliometric analysis for medical research. Indian J Psychol Med. (2023) 45:277–82. 10.1177/02537176221103617 37152388 PMC10159556

[29] Birkle C Pendlebury DA Schnell J Adams J. Web of Science as a data source for research on scientific and scholarly activity. Quantit Sci Stud. (2020) 1:363–76. 10.1162/qss_a_00018

[30] Pasko O Chen F Oriekhova A Brychko A Shalyhina I. Mapping the literature on sustainability reporting: a Bibliometric analysis grounded in Scopus and Web of science core collection. Eur J Sustain Dev. (2021) 10:303–303. 10.14207/ejsd.2021.v10n1p303

[31] Aria M Cuccurullo C. bibliometrix: an R-tool for comprehensive science mapping analysis. J Informetr. (2017) 11:959–75. 10.1016/j.joi.2017.08.007

[32] Van Eck N Waltman L. Software survey: VOSviewer, a computer program for bibliometric mapping. Scientometrics. (2010) 84:523–38. 10.1007/s11192-009-0146-3 20585380 PMC2883932

[33] Chen C. Science mapping: a systematic review of the literature. J Data Inf Sci. (2017) 2:1–40. 10.1515/jdis-2017-0006

[34] Moral-Muñoz JA Herrera-Viedma E Santisteban-Espejo A Cobo MJ. Software tools for conducting bibliometric analysis in science: an up-to-date review. Profesional Inf. (2020) 29:3. 10.3145/epi.2020.ene.0331545655

[35] Stefanis C Stavropoulou E Stavropoulos A Gyriki D Nikolaidis CG Vassos V . Innovative methodological pillars for bibliometric studies: AI screening, data normalization and dual-tool analysis. Discover Appl Sci. (2025) 7:973. 10.1007/s42452-025-07653-3

[36] Fradkin JE Rodgers GP. Diabetes research: a perspective from the National Institute of Diabetes and Digestive and Kidney Diseases. Diabetes. (2013) 62:320–6. 10.2337/db12-0269 23349536 PMC3554357

[37] Cuff DJ Meneilly GS Martin A Ignaszewski A Tildesley HD Frohlich JJ. Effective exercise modality to reduce insulin resistance in women with type 2 diabetes. Diabetes Care. (2003) 26:2977–82. 10.2337/diacare.26.11.2977 14578226

[38] Mitranun W Deerochanawong C Tanaka H Suksom D. Continuous vs. interval training on glycemic control and macro- and microvascular reactivity in type 2 diabetic patients. Scand J Med Sci Sports. (2014) 24:e69–76. 10.1111/sms.12112 24102912

[39] Sigal RJ Kenny GP Boulé NG Wells GA Prud'homme D Fortier M . Effects of aerobic training, resistance training, or both on glycemic control in type 2 diabetes: a randomized trial. Ann Internal Med. (2007) 147:357–69. 10.7326/0003-4819-147-6-200709180-00005 17876019

[40] Committee PAGA. Physical Activity Guidelines Advisory Committee Report, 2008. Washington, DC: US Department of Health and Human Services (2008) 2008:A1–H14.

[41] Riddell MC Gallen IW Smart CE Taplin CE Adolfsson P Lumb AN . Exercise management in type 1 diabetes: a consensus statement. Lancet Diab Endocrinol. (2017) 5:377–90. 10.1016/S2213-8587(17)30014-1 28126459

[42] Maran A Pavan P Bonsembiante B Brugin E Ermolao A Avogaro A . Continuous glucose monitoring reveals delayed nocturnal hypoglycemia after intermittent high-intensity exercise in nontrained patients with type 1 diabetes. Diabetes Technol Ther. (2010) 12:763–8. 10.1089/dia.2010.0038 20807120

[43] Franz MJ MacLeod J Evert A Brown C Gradwell E Handu D . Academy of Nutrition and Dietetics nutrition practice guideline for type 1 and type 2 diabetes in adults: systematic review of evidence for medical nutrition therapy effectiveness and recommendations for integration into the nutrition care process. J Acad Nutr Diet. (2017) 117:1659–79. 10.1016/j.jand.2017.03.022 28533169

[44] Salas-Salvadó J Bulló M Estruch R Ros E Covas MI Ibarrola-Jurado N . Prevention of diabetes with Mediterranean diets: a subgroup analysis of a randomized trial. Ann Intern Med. (2014) 160:1–10. 10.7326/M13-1725 24573661

[45] Van Zuuren EJ Fedorowicz Z Kuijpers T Pijl H. Effects of low-carbohydrate-compared with low-fat-diet interventions on metabolic control in people with type 2 diabetes: a systematic review including GRADE assessments. Am J Clin Nutr. (2018) 108:300–31. 10.1093/ajcn/nqy096 30007275

[46] Nielsen JV Gando C Joensson E Paulsson C. Low carbohydrate diet in type 1 diabetes, long-term improvement and adherence: a clinical audit. Diabetol Metab Syndr. (2012) 4:23. 10.1186/1758-5996-4-23 22650646 PMC3583262

[47] Wheeler ML Dunbar SA Jaacks LM Karmally W Mayer-Davis EJ Wylie-Rosett J . Macronutrients, food groups, and eating patterns in the management of diabetes: a systematic review of the literature, 2010. Diabetes Care. (2012) 35:434–45. 10.2337/dc11-2216 22275443 PMC3263899

[48] Bantle JP Wylie-Rosett J Albright AL Apovian CM Clark NG Franz MJ . Nutrition recommendations and interventions for diabetes: a position statement of the American Diabetes Association. Diabetes Care. (2008) 31:S61–78. 10.2337/dc08-S061 18165339

[49] Franz MJ Bantle JP Beebe CA Brunzell JD Chiasson JL Garg A . Evidence-based nutrition principles and recommendations for the treatment and prevention of diabetes and related complications. Diabetes Care. (2002) 25:148–98. 10.2337/diacare.25.1.148 11772915

[50] Kim HJ Kwon O. Nutrition and exercise: cornerstones of health with emphasis on obesity and type 2 diabetes management–A narrative review. Obesity Rev. (2024) 25:e13762. 10.1111/obr.13762 38715378

[51] Cava E Yeat NC Mittendorfer B. Preserving healthy muscle during weight loss. Adv Nutr. (2017) 8:511–9. 10.3945/an.116.014506 28507015 PMC5421125

[52] Long Y Ye H Yang J Tao X Xie H Zhang J . Effects of a vegetarian diet combined with aerobic exercise on glycemic control, insulin resistance, and body composition: a systematic review and meta-analysis. Eating Weight Disor Stud Anorexia, Bulimia Obesity. (2023) 28:9. 10.1007/s40519-023-01536-5 36790517 PMC9931794

[53] Legaard GE Lyngbæk MP Almdal TP Karstoft K Bennetsen SL Feineis CS . Effects of different doses of exercise and diet-induced weight loss on beta-cell function in type 2 diabetes (DOSE-EX): a randomized clinical trial. Nat Metab. (2023) 5:880–95. 10.1038/s42255-023-00799-7 37127822 PMC10229430

[54] Verboven K Wens I Vandenabeele F Stevens A Celie B Lapauw B . Impact of exercise-nutritional state interactions in patients with type 2 diabetes. Med Sci Sports Exerc. (2020) 52:720–8. 10.1249/MSS.0000000000002165 31652237

[55] Colberg SR. Nutrition and exercise performance in adults with type 1 diabetes. Canadian J Diabetes. (2020) 44:750–8. 10.1016/j.jcjd.2020.05.014 32847769

[56] Gomez AM Gomez C Aschner P Veloza A Muñoz O Rubio C . Effects of performing morning versus afternoon exercise on glycemic control and hypoglycemia frequency in type 1 diabetes patients on sensor-augmented insulin pump therapy. J Diabetes Sci Technol. (2015) 9:619–24. 10.1177/1932296814566233 25555390 PMC4604526

[57] Turner D Luzio S Gray B Dunseath G Rees E Kilduff L . Impact of single and multiple sets of resistance exercise in type 1 diabetes. Scand J Med Sci Sports. (2015) 25:e99–e109. 10.1111/sms.12202 24646137

[58] Colberg SR Sigal RJ Fernhall B Regensteiner JG Blissmer BJ Rubin RR . Exercise and type 2 diabetes: the American College of Sports Medicine and the American Diabetes Association: joint position statement. Diabetes Care. (2010) 33:e147–67. 10.2337/dc10-9990 21115758 PMC2992225

[59] Cohen N Dunstan D Robinson C Vulikh E Zimmet P Shaw J. Improved endothelial function following a 14-month resistance exercise training program in adults with type 2 diabetes. Diabetes Res Clin Pract. (2008) 79:405–11. 10.1016/j.diabres.2007.09.020 18006170

[60] Zoppini G Targher G Zamboni C Venturi C Cacciatori V Moghetti P . Effects of moderate-intensity exercise training on plasma biomarkers of inflammation and endothelial dysfunction in older patients with type 2 diabetes. Nutr Metab Cardiov Dis. (2006) 16:543–9. 10.1016/j.numecd.2005.09.004 17126770

[61] Pena KE Stopka CB Barak S Gertner Jr HR Carmeli E. Effects of low-intensity exercise on patients with peripheral artery disease. Phys Sportsmed. (2009) 37:106–10. 10.3810/PSM.2009.04.1689 20048494

[62] Bo S Ciccone G Rosato R Gancia R Grassi G Merletti F . Renal damage in patients with Type 2 diabetes: a strong predictor of mortality. Diabetic Med. (2005) 22:258–65. 10.1111/j.1464-5491.2004.01394.x 15717872

[63] Azadbakht L Atabak S Esmaillzadeh A. Soy protein intake, cardiorenal indices, and C-reactive protein in type 2 diabetes with nephropathy: a longitudinal randomized clinical trial. Diabetes Care. (2008) 31:648–54. 10.2337/dc07-2065 18184902

[64] Albright A Franz M Hornsby G Kriska A Marrero D Ullrich I . American College of Sports Medicine position stand. Exercise and type 2 diabetes. Med Sci Sports Exerc. (2000) 32:1345–60. 10.1097/00005768-200007000-00024 10912903

[65] Ren C Liu W Li J Cao Y Xu J Lu P. Physical activity and risk of diabetic retinopathy: a systematic review and meta-analysis. Acta Diabetol. (2019) 56:823–37. 10.1007/s00592-019-01319-4 30900027

[66] Vinik AI Ziegler D. Diabetic cardiovascular autonomic neuropathy. Circulation. (2007) 115:387–97. 10.1161/CIRCULATIONAHA.106.634949 17242296

[67] Didangelos T Karlafti E Kotzakioulafi E Margariti E Giannoulaki P Batanis G . Vitamin B12 supplementation in diabetic neuropathy: a 1-year, randomized, double-blind, placebo-controlled trial. Nutrients. (2021) 13:395. 10.3390/nu13020395 33513879 PMC7912007

[68] Zilliox LA Russell JW. Physical activity and dietary interventions in diabetic neuropathy: a systematic review. Clin Auton Res. (2019) 29:443–55. 10.1007/s10286-019-00607-x 31076938 PMC6697618

[69] Hinder LM O'Brien PD Hayes JM Backus C Solway AP Sims-Robinson C . Dietary reversal of neuropathy in a murine model of prediabetes and metabolic syndrome. Dis Model Mech. (2017) 10:717–25. 10.1242/dmm.028530 28381495 PMC5483005

[70] Evans WJ Cyr-Campbell D. Nutrition, exercise, and healthy aging. J Am Diet Assoc. (1997) 97:632–8. 10.1016/S0002-8223(97)00160-0 9183325

[71] Fleg JL. Aerobic exercise in the elderly: a key to successful aging. Discov Med. (2012) 13:223–8. 22463798

[72] Hunter GR McCarthy JP Bamman MM. Effects of resistance training on older adults. Sports Med. (2004) 34:329–48. 10.2165/00007256-200434050-00005 15107011

[73] Peterson MD Sen A Gordon PM. Influence of resistance exercise on lean body mass in aging adults: a meta-analysis. Med Sci Sports Exerc. (2011) 43:249. 10.1249/MSS.0b013e3181eb6265 20543750 PMC2995836

[74] Beaudry KM Devries MC. Nutritional strategies to combat type 2 diabetes in aging adults: the importance of protein. Front Nutr. (2019) 6:138. 10.3389/fnut.2019.00138 31555655 PMC6724448

[75] Mithal A Bonjour JP Boonen S Burckhardt P Degens H El Hajj Fuleihan G . Impact of nutrition on muscle mass, strength, and performance in older adults. Osteop Int. (2013) 24:1555–66. 10.1007/s00198-012-2236-y 23247327

[76] Cermak NM de Groot LC Saris WH Van Loon LJ. Protein supplementation augments the adaptive response of skeletal muscle to resistance-type exercise training: a meta-analysis. Am J Clin Nutr. (2012) 96:1454–64. 10.3945/ajcn.112.037556 23134885

[77] Tieland M Dirks ML van der Zwaluw N Verdijk LB Van De Rest O de Groot LC . Protein supplementation increases muscle mass gain during prolonged resistance-type exercise training in frail elderly people: a randomized, double-blind, placebo-controlled trial. J Am Med Dir Assoc. (2012) 13:713–9. 10.1016/j.jamda.2012.05.020 22770932

[78] Gao L Peng W Xue H Wu Y Zhou H Jia P . Spatial–temporal trends in global childhood overweight and obesity from 1975 to 2030: a weight mean center and projection analysis of 191 countries. Global Health. (2023) 19:53. 10.1186/s12992-023-00954-5 37542334 PMC10403851

[79] Akhuemonkhan E Lazo M. Association between family history of diabetes and cardiovascular disease and lifestyle risk factors in the United States population: the 2009-2012 National Health and Nutrition Examination Survey. Prevent Med. (2017) 96:129–34. 10.1016/j.ypmed.2016.12.015 28007493

[80] Hasbullah FY Fong KY Ismail A Mitri J. Yusof BNM a comparison of nutritional status, knowledge and type 2 diabetes risk among Malaysian young adults with and without family history of diabetes. Malaysian J Med Sci. (2021) 28:75–86. 10.21315/mjms2021.28.1.10 33679223 PMC7909351

[81] Moon JH Roh E Oh TJ Kim KM Moon JH Lim S . Increased risk of metabolic disorders in healthy young adults with family history of diabetes: from the Korea National Health and Nutrition Survey. Diabetol Metab Syndr. (2017) 9:16. 10.1186/s13098-017-0210-8 28265302 PMC5333414

[82] Daniels SR Arnett DK Eckel RH Gidding SS Hayman LL Kumanyika S . Overweight in children and adolescents: pathophysiology, consequences, prevention, and treatment. Circulation. (2005) 111:1999–2012. 10.1161/01.CIR.0000161369.71722.10 15837955

[83] Davis CL Pollock NK Waller JL Allison JD Dennis BA Bassali R . Exercise dose and diabetes risk in overweight and obese children: a randomized controlled trial. Jama. (2012) 308:1103–12. 10.1001/2012.jama.10762 22990269 PMC3487697

[84] Organization WH. Diet, Nutrition, and the Prevention of Chronic Diseases: Report of a Joint WHO/FAO Expert Consultation. Geneva: World Health Organization. (2003).

[85] Baker JL Farpour-Lambert NJ Nowicka P Pietrobelli A Weiss R. Evaluation of the overweight/obese child–practical tips for the primary health care provider: recommendations from the Childhood Obesity Task Force of the European Association for the Study of Obesity. Obes Facts. (2010) 3:131–7. 10.1159/000295112 20484947 PMC6452124

[86] Giannini C de Giorgis T Mohn A Chiarelli F. Role of physical exercise in children and adolescents with diabetes mellitus. J Pediatr Endocrinol Metabol. (2007) 20:173–84. 10.1515/JPEM.2007.20.2.173 17396433

[87] Smart CE Annan F Bruno LP Higgins LA Acerini CL. Nutritional management in children and adolescents with diabetes. Pediatr Diab. (2014) 15:100–117. 10.1111/pedi.12175 25182313

[88] Nakshine VS Jogdand SD. A comprehensive review of gestational diabetes mellitus: impacts on maternal health, fetal development, childhood outcomes, and long-term treatment strategies. Cureus. (2023) 15:47500. 10.7759/cureus.47500 38021940 PMC10663705

[89] Ratner RE Christophi CA Metzger BE Dabelea D Bennett PH Pi-Sunyer X . Prevention of diabetes in women with a history of gestational diabetes: effects of metformin and lifestyle interventions. J Clin Endocrinol Metab. (2008) 93:4774–9. 10.1210/jc.2008-0772 18826999 PMC2626441

[90] Marshall NE Abrams B Barbour LA Catalano P Christian P Friedman JE . The importance of nutrition in pregnancy and lactation: lifelong consequences. Am J Obstet Gynecol. (2022) 226:607–32. 10.1016/j.ajog.2021.12.035 34968458 PMC9182711

[91] Committee ADAPP. 15 Management of diabetes in pregnancy: standards of care in diabetes–2025. Diabetes Care. (2025) 48:S306–S320. 10.2337/dc25-S015 39651985 PMC11635054

[92] Rogozińska E Chamillard M Hitman GA Khan KS Thangaratinam S. Nutritional manipulation for the primary prevention of gestational diabetes mellitus: a meta-analysis of randomised studies. PLoS ONE. (2015) 10:e0115526. 10.1371/journal.pone.0115526 25719363 PMC4342242

[93] Allehdan SS Basha AS Asali FF Tayyem RF. Dietary and exercise interventions and glycemic control and maternal and newborn outcomes in women diagnosed with gestational diabetes: systematic review. Diab Metab Syndr. (2019) 13:2775–84. 10.1016/j.dsx.2019.07.040 31405707

[94] DiPietro L Evenson KR Bloodgood B Sprow K Troiano RP Piercy KL . Benefits of physical activity during pregnancy and postpartum: an umbrella review. Med Sci Sports Exerc. (2019) 51:1292. 10.1249/MSS.0000000000001941 31095086 PMC6527310

[95] Padayachee C Coombes JS. Exercise guidelines for gestational diabetes mellitus. World J Diabetes. (2015) 6:1033. 10.4239/wjd.v6.i8.1033 26240700 PMC4515443

[96] Artal R O'Toole M. Guidelines of the American College of Obstetricians and Gynecologists for exercise during pregnancy and the postpartum period. Br J Sports Med. (2003) 37:6–12. 10.1136/bjsm.37.1.6 12547738 PMC1724598

[97] Lloyd-Jones DM Allen NB Anderson CA Black T Brewer LC Foraker RE . Life's essential 8: updating and enhancing the American Heart Association's construct of cardiovascular health: a presidential advisory from the American Heart Association. Circulation. (2022) 146:e18–43. 10.1161/CIR.0000000000001078 35766027 PMC10503546

[98] Hare MJ Barzi F Boyle JA Guthridge S Dyck RF Barr EL . Diabetes during pregnancy and birthweight trends among Aboriginal and non-Aboriginal people in the Northern Territory of Australia over 30 years. Lancet Reg Health Western Pacific. (2020) 1:100005. 10.1016/j.lanwpc.2020.100005 34327339 PMC8315488

[99] Kirkham R King S Graham S Boyle J Whitbread C Skinner T . ‘No sugar', ‘no junk food', ‘do more exercise'–moving beyond simple messages to improve the health of Aboriginal women with Hyperglycaemia in Pregnancy in the Northern Territory–A phenomenological study. Women and Birth. (2021) 34:578–84. 10.1016/j.wombi.2020.10.003 33144033

[100] Dias T MacKay D Canuto K Boyle JA D'Antoine H Hampton D . Supporting healthy lifestyles for First Nations women and communities through co-design: lessons and early findings from remote Northern Australia. Front Clin Diab Healthcare. (2024) 5:1356060. 10.3389/fcdhc.2024.1356060 38863516 PMC11165116

[101] Wolever R Dreusicke M Fikkan J Hawkins T Yeung S Wakefield J . Integrative health coaching for patients with type 2 diabetes. Diabetes Educ. (2010) 36:629–39. 10.1177/0145721710371523 20534872

[102] He J Chu N Wan H Ling J Xue YC Leung K . Use of technology in prediabetes and precision prevention. J Diabetes Investig. (2025) 16:1217–31. 10.1111/jdi.70057 40317994 PMC12209524

[103] Hietbrink EAG Nijeweme-d'Hollosy WO Middelweerd A Konijnendijk AAJ Schrijver LK Voorde AST . A digital coach (E-supporter 1.0) to support physical activity and a healthy diet in people with type 2 diabetes: acceptability and limited efficacy testing. JMIR Form Res. (2023) 7:e45294. 10.2196/45294 37505804 PMC10422172

[104] Hietbrink EAG Middelweerd A van Empelen P Preuhs K Konijnendijk AAJ Nijeweme-d'Hollosy WO . A digital lifestyle coach (e-supporter 1.0) to support people with type 2 diabetes: participatory development study. JMIR Hum Factors. (2023) 10:e40017. 10.2196/40017 36633898 PMC9947918

[105] O'Donovan G Lee IM Hamer M Stamatakis E. Association of “weekend warrior” and other leisure time physical activity patterns with risks for all-cause, cardiovascular disease, and cancer mortality. JAMA Intern Med. (2017) 177:335–42. 10.1001/jamainternmed.2016.8014 28097313

[106] Chen Z Jia J Tu J Zhao Y Li X. Association between diabetes prevalence and weekend warrior activity patterns. Public Health. (2025) 240:97–103. 10.1016/j.puhe.2025.01.016 39892018

[107] Mahe J Xu A Liu L Hua L Tu H Huo Y . Association between weekend warrior physical activity pattern and all-cause mortality among adults living with type 2 diabetes: a prospective cohort study from NHANES 2007 to 2018. Diabetol Metab Syndr. (2024) 16:226. 10.1186/s13098-024-01455-0 39267148 PMC11391736

[108] O'Donovan G Sarmiento OL Hamer M. The rise of the “weekend warrior”. J Orthop Sports Phys Ther. (2018) 48:604–6. 10.2519/jospt.2018.0611 30064334

