# Supplementary material for: Mapping the research trends and hotspots of exercise and nutrition in diabetes: a bibliometric and visual analysis (2005–2025)
Source: Front Nutr. 2025 Oct 9;12:1680190. doi: 10.3389/fnut.2025.1680190 (PMC12545082; doi:10.3389/fnut.2025.1680190)
Supplement: Supplementary file 1 [file Data_Sheet_1.pdf]

SUPPLEMENTARY MATERIAL

SUPPLEMENTARY TABLES

Table 1. Search strategy and inclusion criteria for literature retrieved from WoSCC.

Parameter	Description
Database	Web of Science Core Collection (WoSCC)
Retrieval Date	July 3, 2025
Search Field	Topic Search (TS)
Search Terms	diabetes AND (exercise OR “physical activity”) AND nutrition*
Publication period	January 1, 2005, to July 3, 2025
Document Types	Article or Review
Language	English only
Data Cleaning	Irrelevant and duplicate documents were excluded
Results	4,793 publications retrieved
Data Export	Exported in plain text format with full records and cited references

Note: TS = Topic Search (Title, Abstract, Author Keywords, Keywords Plus); quotation marks (“”) retrieve exact phrases; asterisk (*) represents any group of characters, including none.

Table 2. Search strategy and inclusion criteria for literature retrieved from Scopus.

Parameter	Description
Database	Scopus
Retrieval Date	July 3, 2025
Search Field	Title/Abstract/Keywords
Search Terms	diabetes AND (exercise OR “physical activity”) AND nutrition*
Publication period	January 1, 2005, to July 3, 2025
Document Types	Article or Review
Language	English only
Data Cleaning	Irrelevant and duplicate documents were excluded
Results	9,187 publications retrieved
Data Export	Exported in CSV format with full records and cited references

SUPPLEMENTARY FIGURES

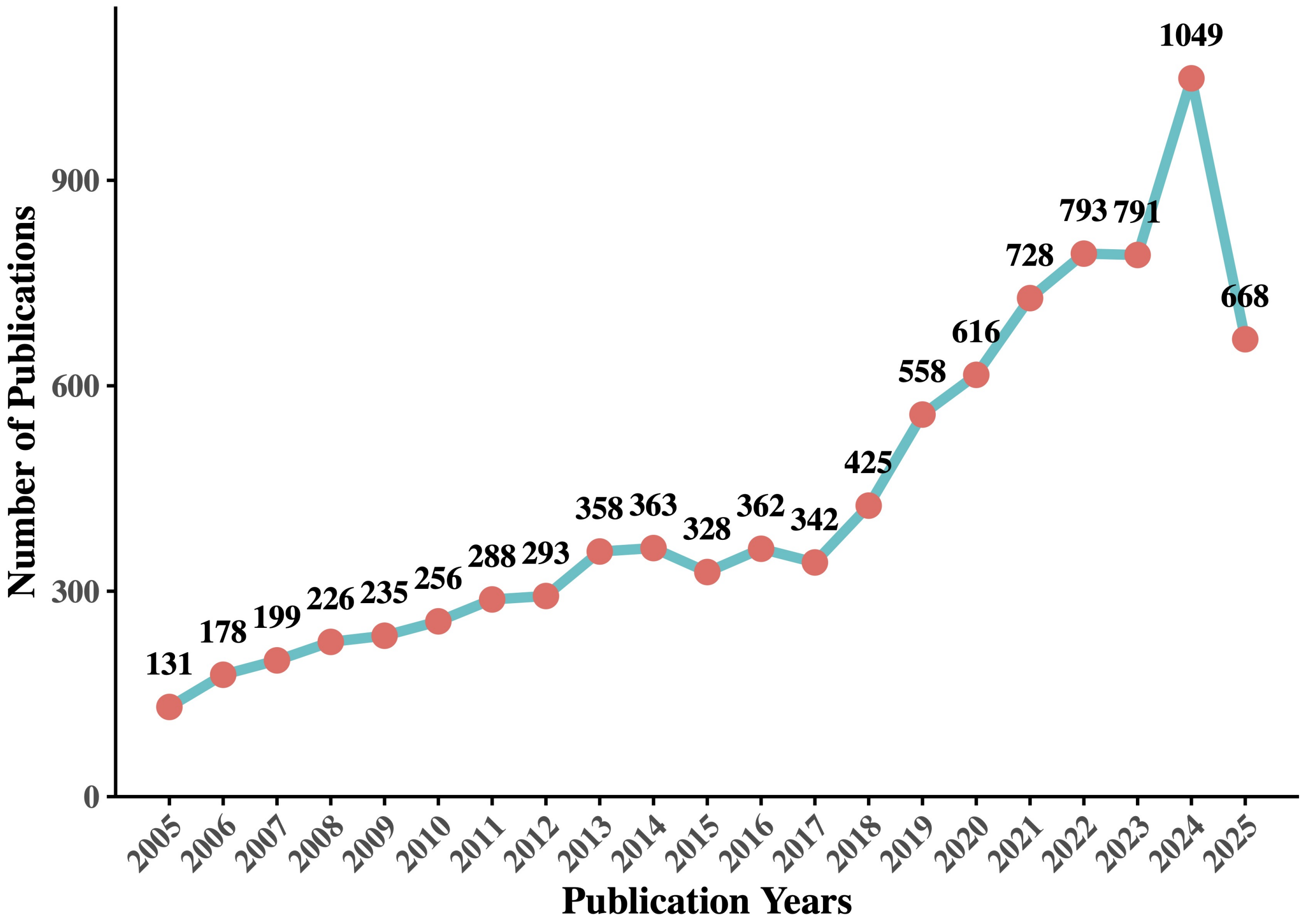

Figure 1. Trends of annual publication outputsfrom the Scopus database.

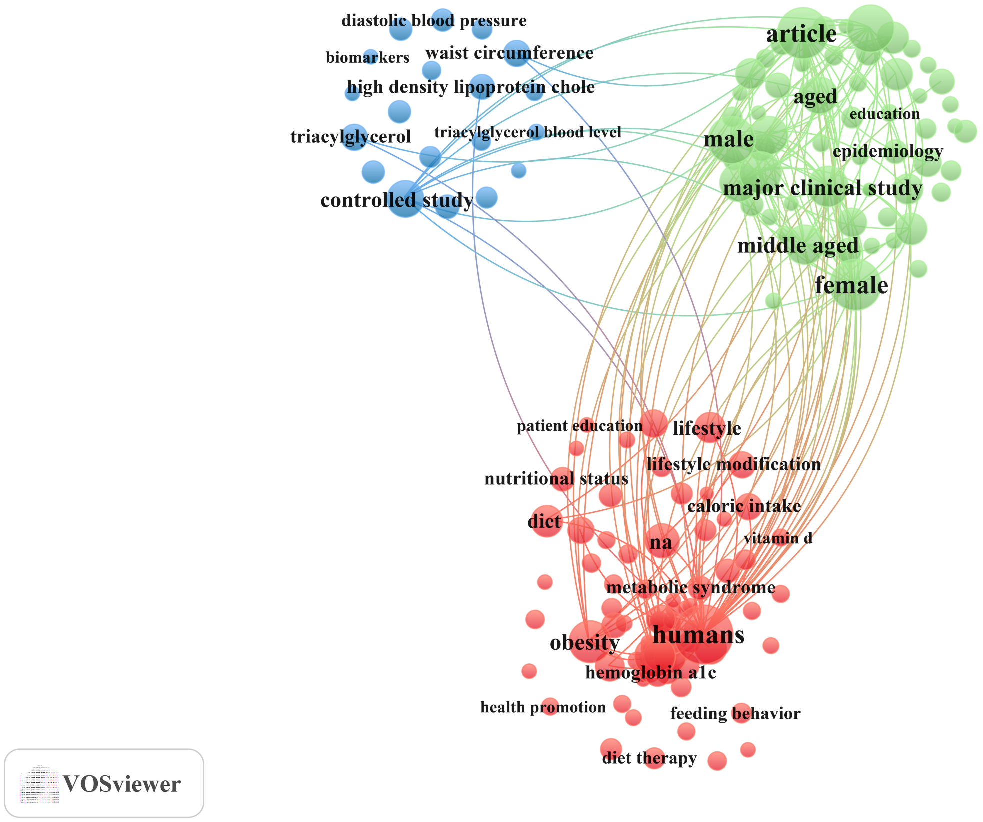

Figure 2. Keyword co-occurrence map of publications on exercise and nutrition in diabetes from the Scopus database.
